# Digital Papillary Adenocarcinoma: A Case Report

**DOI:** 10.7759/cureus.95313

**Published:** 2025-10-24

**Authors:** Farlin Asharaff, Jeffery Theaker, Geeta Belgi

**Affiliations:** 1 Dermatology, University Hospital Southampton NHS Foundation Trust, Southampton, GBR; 2 Histopathology, University Hospital Southampton NHS Foundation Trust, Southampton, GBR

**Keywords:** amputation, biopsy, case report, digital papillary adenocarcinoma, histopathology

## Abstract

We present the case of a 58-year-old male with a five-year history of a solitary, recurrent nodule on the distal pulp of his right middle finger. Histological examination confirmed the diagnosis of digital papillary adenocarcinoma (DPA). Given the lesion’s recurrence and bone involvement, amputation of the terminal phalanx was performed following multidisciplinary team (MDT) discussion.

Postoperatively, the patient entered a structured surveillance program, consisting of clinical reviews every six months and annual CT imaging of the chest, abdomen, and pelvis to monitor for recurrence or metastasis. At three years post-amputation, the patient remains clinically well with no evidence of disease recurrence. This case highlights the importance of a multidisciplinary approach and long-term follow-up in managing DPA, given its potential for local recurrence and distant metastasis.

## Introduction

Skin adnexal tumors are primary cutaneous neoplasms that originate from adnexal structures such as hair follicles, sebaceous glands, and sweat glands. These tumors encompass a wide spectrum of histological subtypes, with most being benign. However, malignant variants can occur, often posing diagnostic challenges due to their diverse clinical and morphological features [1]. 

Digital papillary adenocarcinoma (DPA) is a rare, malignant adnexal tumor that arises from the eccrine sweat glands, most commonly affecting the digits. It was first described in literature by Helwig et al. in 1984; they described it as an eccrine acrospiroma [2]. Acral regions are the most affected areas, especially the volar aspect of the finger, but DPA has been reported to be in other regions such as the ankle, genitalia, etc. [3]. It has been commonly reported in Caucasian men above the age of 50 [4]. Well-differentiated variants of DPA can be easily mistaken for benign sweat gland tumors such as nodular hidradenoma, due to overlapping histological features. Accurate diagnosis, therefore, relies heavily on detailed histopathological and immunohistochemical evaluation [5].

Given the rarity of these tumors, there are currently no established clinical guidelines regarding optimal surgical excision margins or standardized management protocols. Long-term follow-up is essential, as DPA carries a significant risk of local recurrence and distant metastasis, even several years after initial excision [6]. 

## Case presentation

The patient is a 58-year-old male who presented with a five-year history of a recurrent lesion on his right middle finger. He recalled a history of trauma to the finger several years prior, having crushed it in a door. Following the injury, he developed a hemorrhagic lesion, which was surgically excised. However, the lesion recurred at the same site and became intermittently inflamed over time.

Clinical examination

**Figure 1:**
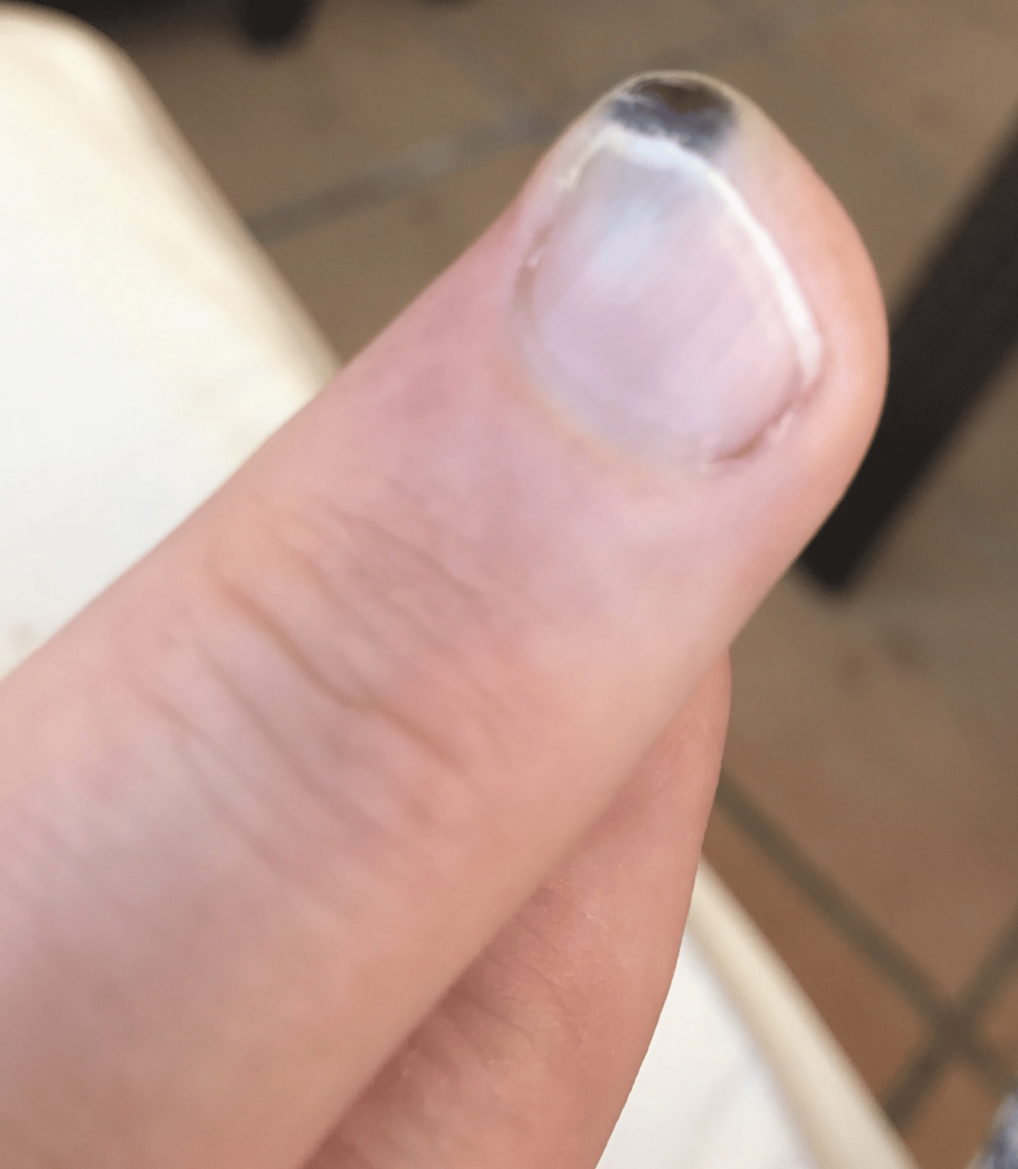
Smooth swelling located over the distal ulnar aspect of the right middle finger

**Figure 2:**
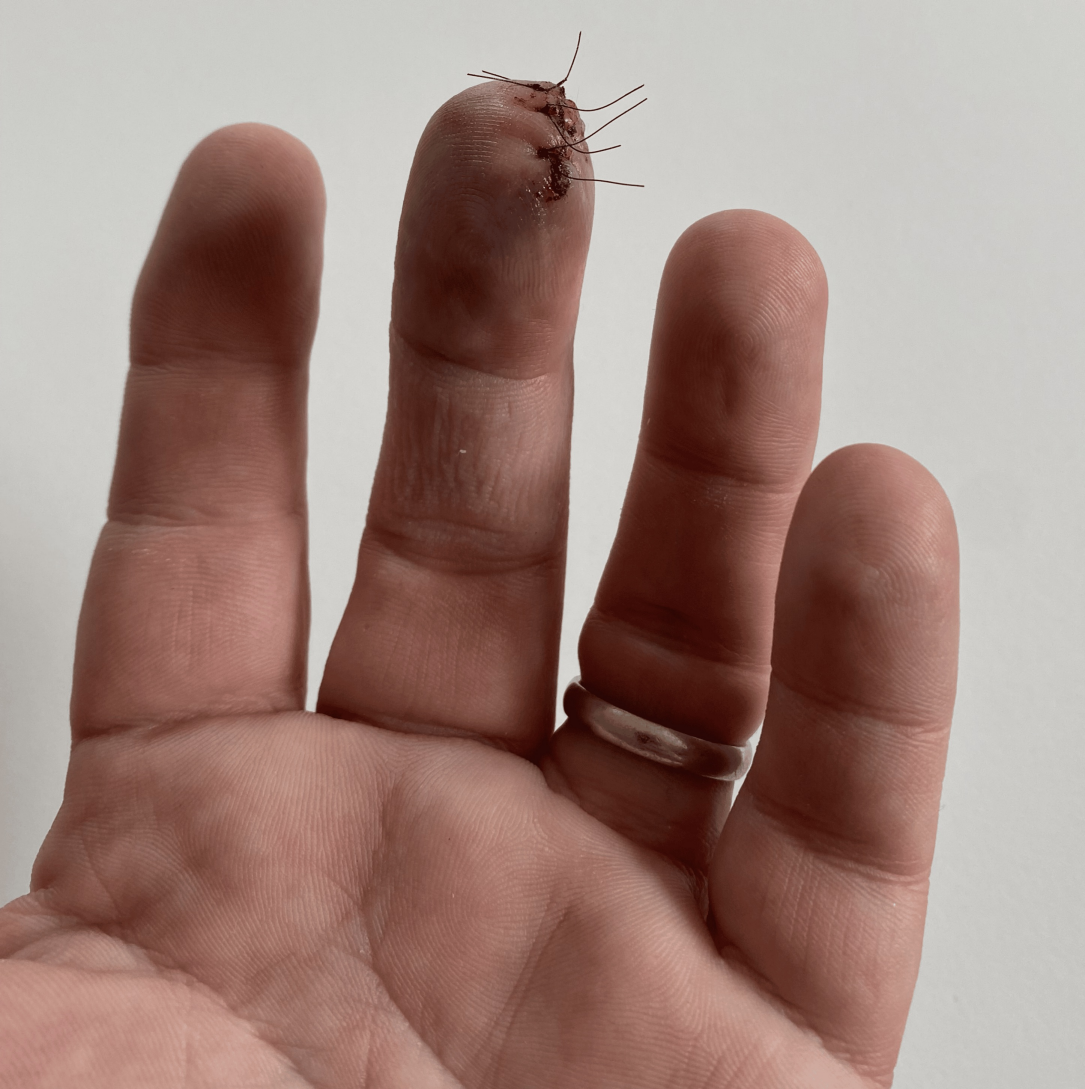
: Post-biopsy site

On examination, there was a 1 cm, non-tender, smooth lesion located over the distal ulnar aspect of the pulp of the right middle finger. The lesion was eroding the underlying distal phalanx (Figure 1). A biopsy was performed, producing multiple small fragments (Figure 2).

Histopathological findings

**Figure 3:**
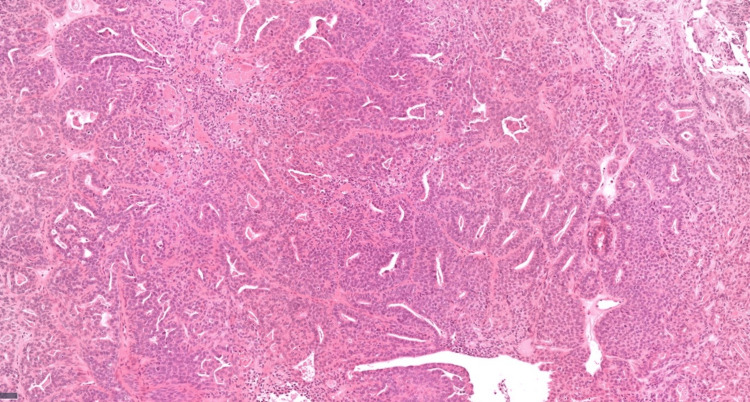
Medium power histology image (x10) showing a solid and glandular epithelial tumour

**Figure 4:**
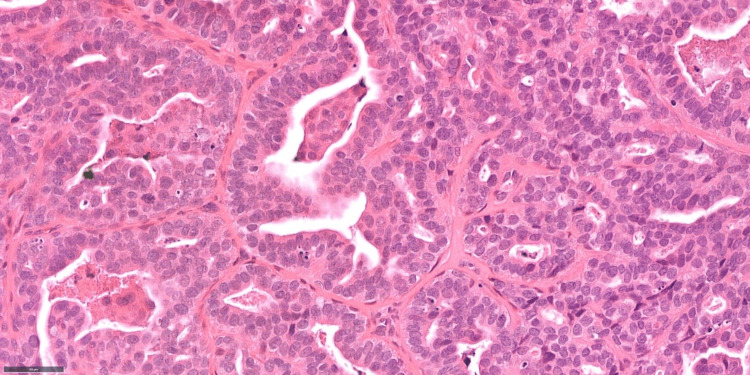
High power histology image (x40) with glandular and papillary areas showing cytological atypia and mitotic activity

Histological examination revealed a moderately differentiated adenocarcinoma exhibiting solid, glandular, and papillary architectural patterns (Figures 3-4), with evidence of invasion into the surrounding fibrous stroma. In certain areas, a double-layered arrangement was observed, indicative of both epithelial and myoepithelial differentiation. Immunohistochemical analysis demonstrated positivity for epithelial and myoepithelial markers, including cytokeratin 7 (CK7), epithelial membrane antigen (EMA), S100, carcinoembryonic antigen (CEA), smooth muscle actin (SMA), and p63.

**Figure 5:**
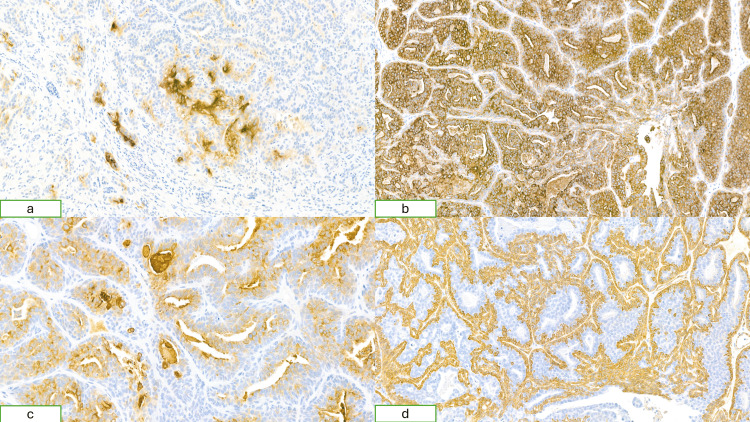
Histopathological image (a) CEA shows expression in luminal cells, consistent with sweat gland differentiation; (b) CK7 demonstrates diffuse expression throughout the tumor; (c) EMA is expressed in the luminal cells but absent in the outer myoepithelial layer; (d) SMA shows expression in the outer myoepithelial cells with no staining in the inner luminal cells CEA: Carcinoembryonic antigen, CK7: Cytokeratin 7, EMA: Epithelial membrane antigen, SMA: Smooth muscle actin

The luminal cells showed positive staining for CEA, consistent with sweat gland differentiation, while CK7 showed diffuse expression throughout the tumor. The outer myoepithelial cells were positive for SMA and negative for EMA, whereas EMA was expressed in the luminal cells but absent in the outer myoepithelial layer (Figure 5).

These findings were consistent with a diagnosis of a primary sweat gland tumor and more specifically in keeping with DPA. There was no evidence of perineural or vascular invasion. Due to concerns about incomplete excision in this fragmented specimen, a distal amputation of the right middle finger was advised by the multidisciplinary team (MDT).

Management and follow-up

**Figure 6:**
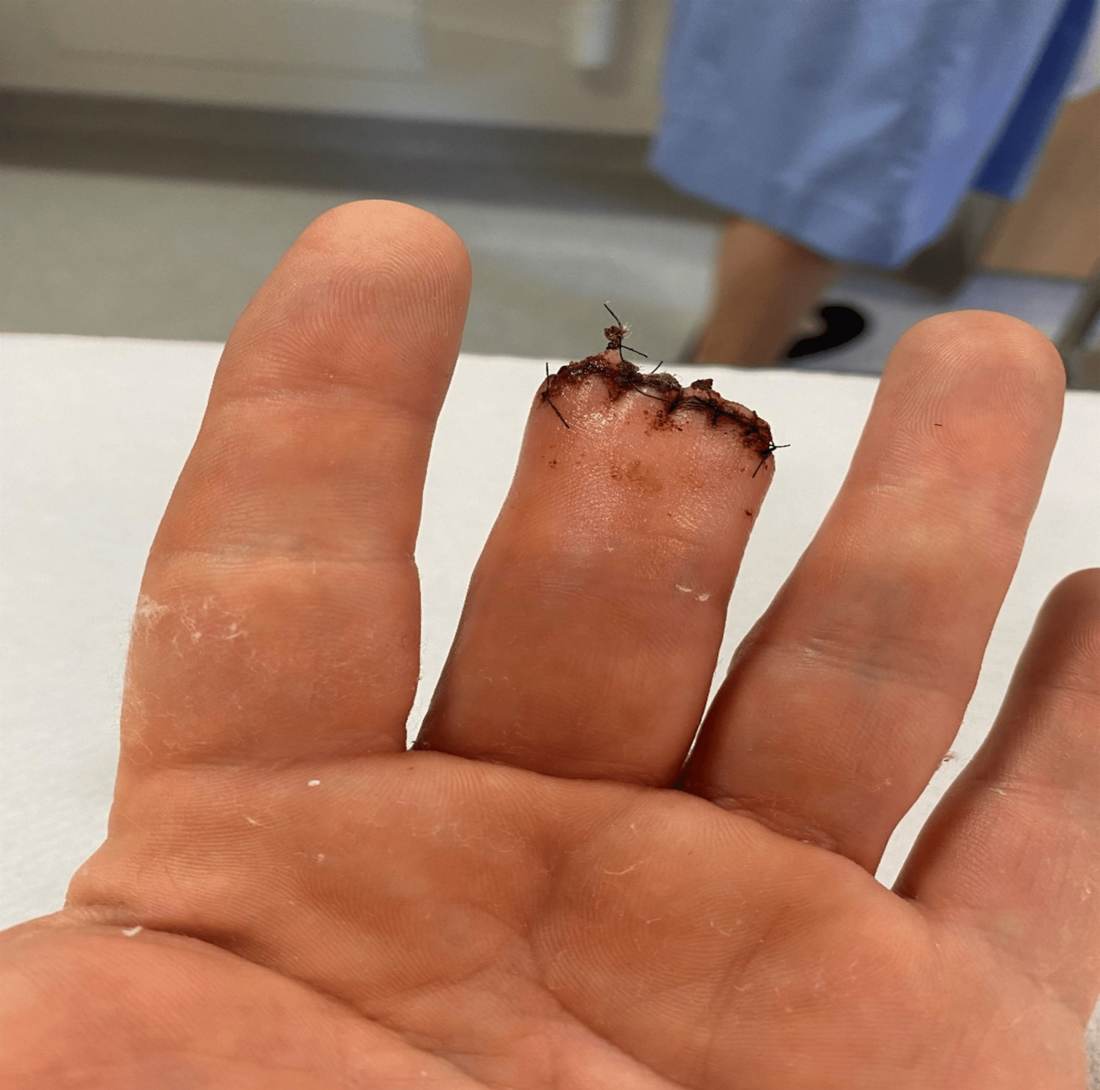
Post-amputation site

One month later, the patient underwent terminal phalanx amputation (Figure 6). Histological examination of the excised specimen revealed no residual tumor in the subcutis or bone and evidence of post-surgical changes only. The case was discussed in a skin cancer MDT, which included input from oncologists, plastic and maxillofacial surgeons, and dermatologists. The team recommended long-term surveillance due to the high recurrence rate associated with DPA. The follow-up protocol included clinical review every six months and annual CT scans of the neck, chest, abdomen, and pelvis. Over the past three years, since amputation, the patient has undergone three staging CT scans of the thorax, abdomen, and pelvis, all of which have been unremarkable. He remains clinically well and under vigilant follow-up.

## Discussion

Digital papillary adenocarcinoma is a rare malignant eccrine sweat gland tumor, most frequently presenting on the digits. Due to its rarity, there are currently no standardized, evidence-based guidelines for surgical excision margins or overall management strategy [6]. Multiple surgical approaches have been reported in the literature, including wide local excision (WLE), digit amputation, and Mohs micrographic surgery (MMS). A systematic review published in March 2025 reported zero recurrences following MMS, suggesting it may be the most effective option in achieving complete tumor clearance. In contrast, WLE and digit amputation were associated with recurrence rates of 34.1% and 20%, respectively [7]. However, in our case, digit amputation proves to be an effective therapeutic intervention, as no local recurrence or distant metastasis has been observed during the three years of follow-up. This is supported by Mudduwa et al., as they recommend the use of sentinel lymph node biopsy (SLNB) and radical excision or amputation as optimal interventions for the management of DPA [8]. 

Interestingly, a 2023 case series revealed that DPA can occur in non-acral locations and demonstrated a strong association with human papillomavirus (HPV) type 42, as next-generation sequencing identified HPV42 in all cases [9]. Vanderbilt et al. illustrated that integrating histopathological evaluation with immunohistochemical profiling and HPV42 detection may minimize diagnostic errors and facilitate accurate differentiation of DPA from other neoplasms [5]. However, HPV status was not assessed in our patient.

The aggressive behavior of DPA has long been recognized. Duke et al. coined the term 'aggressive digital papillary adenocarcinoma' in 2000 to highlight its high risk of recurrence and metastatic potential [10]. This was reinforced by Kao et al., who proposed that aggressive DPA and adenocarcinoma should be categorized as distinct clinicopathological entities due to their malignant nature [4].

Current evidence suggests limited efficacy of chemotherapy in the metastatic setting, with most case reports indicating poor response [6]. Notably, a 2016 case report described the only documented instance of complete tumor regression following five weeks of radiotherapy combined with surgical excision, highlighting radiotherapy as a potential adjunct in select cases [11]. Emerging molecular studies have furthered our understanding of DPA. A 2019 transcriptomic analysis demonstrated fibroblast growth factor receptor 2 (FGFR2) overexpression in DPA, indicating that targeting the fibroblast growth factor (FGF)/FGFR signalling axis may represent a promising future therapeutic strategy [12].

## Conclusions

In our case, amputation of the terminal phalanx was performed due to underlying bone involvement and concerns regarding incomplete excision with more conservative approaches. At the three-year follow-up, the patient remains free of local recurrence or distant metastasis, supporting digital amputation as a potentially effective treatment option when achieving clear margins is critical. We aim to contribute to the growing body of literature on DPA by highlighting the success of surgical amputation in our patient and reinforcing the importance of MDT management and long-term surveillance in this rare but aggressive tumor.
